# Restless legs syndrome in chronic myeloid leukemia: an overlooked condition with a significant impact on health-related quality of life

**DOI:** 10.1007/s00277-026-06832-5

**Published:** 2026-03-19

**Authors:** Turgut Gürer, Ulgar Boran Günay, Berkay Kılıç, Ayşegül Akkan Suzan, Duygu Seyhan Erdoğan, Deniz Özmen, Tuğrul Elverdi, Ayşe Salihoğlu, Ayşegül Gündüz, Muhlis Cem Ar, Zafer Başlar, Ahmet Emre Eşkazan

**Affiliations:** 1 Department of Internal Medicine, Cerrahpaşa Faculty of Medicine, Istanbul University-Cerrahpaşa, Istanbul, Turkey; 2 Cerrahpaşa Faculty of Medicine, Istanbul University-Cerrahpaşa, Istanbul, Turkey; 3 Division of Clinical Neurophysiology, Department of Neurology, Cerrahpaşa Faculty of Medicine, Istanbul University-Cerrahpaşa, Istanbul, Turkey; 4 Department of Public Health, Division of Occupational Medicine, Gazi University, Ankara, Turkey; 5 Division of Hematology, Department of Internal Medicine, Cerrahpaşa Faculty of Medicine, Istanbul University-Cerrahpaşa, Istanbul, Turkey

**Keywords:** Chronic myeloid leukemia, Quality of life, Restless legs syndrome, Tyrosine kinase inhibitor, Symptom burden

## Abstract

Restless legs syndrome (RLS) is a common but often overlooked cause of sleep disturbance and discomfort during rest. The prevalence of RLS remains unknown in chronic myeloid leukemia (CML). This study explores its frequency, clinical relevance, and impact on quality of life in patients with CML. We consecutively evaluated CML patients who visited our outpatient clinic between January and August 2023. Patients with iron deficiency (ID) and healthy subjects (HS) were also included as controls. Standardized psychometric assessments were administered to CML patients, including IRLSSG Rating Scale, Beck inventories, PSQI, FACIT-Fatigue, and EORTC QLQ-C30/CML24 for evaluating health-related quality of life (HRQoL). Neurological examinations and electromyography were performed in all participants diagnosed with RLS, including controls, to evaluate for secondary causes of peripheral polyneuropathy. A total of 164 CML patients, 22 individuals with ID, and 23 HSs were included. The rates of RLS were 20.1% and 45.5% in patients with CML and ID, respectively. None of the HS had RLS. Compared to non-RLS CML patients, those with RLS exhibited significantly poorer sleep quality (p = 0.016), greater anxiety (p < 0.001), depression (p = 0.005), and fatigue (p < 0.001), alongside elevated symptom burden scores (p < 0.001). Molecular responses and TKI dose modifications were similar between CML patients with and without RLS. Not using ACEi/ARB was associated with a 0.235-fold reduced risk of RLS, and a higher symptom burden score was independently associated with RLS (OR = 1.077). RLS is an underrecognized comorbidity in CML that severely impairs HRQoL, despite no impact on treatment response. Routine screening and targeted symptom management strategies are warranted.

**Supplementary Information:**

The online version contains supplementary material available at 10.1007/s00277-026-06832-5.

## Introduction

Myeloproliferative neoplasms (MPNs) are a group of clonal hematopoietic stem cell disorders characterized by excessive proliferation of myeloid lineages in the bone marrow, resulting in sustained elevations in peripheral blood cell counts. Chronic myeloid leukemia (CML), a classical MPN, is defined by the presence of the Philadelphia chromosome resulting from a t(9;22)(q34;q11) translocation, which gives rise to the *BCR::ABL1* fusion oncogene [1, 2]. Tyrosine kinase inhibitors (TKIs) targeting BCR::ABL1 have revolutionized CML therapy, transforming it into a chronic condition with near-normal life expectancy for many patients [3]. Consequently, attention has increasingly turned toward long-term survivorship issues, including health-related quality of life (HRQoL), psychiatric comorbidities, and neurologic complications.

Restless legs syndrome (RLS), also known as Willis–Ekbom disease, is a common yet underdiagnosed sensorimotor disorder, characterized by an uncontrollable urge to move the legs, typically during periods of rest and in the evening [4]. Although the precise pathophysiology remains unclear, iron metabolism dysfunction, dopaminergic system abnormalities, and peripheral neuropathies have been implicated in the condition. A growing body of evidence suggests that RLS is more prevalent in patients with Philadelphia-negative MPNs, such as polycythemia vera and essential thrombocythemia, than in the general population [5–7]. However, no study to date has investigated the prevalence of RLS in CML or its potential impact on HRQoL or disease outcomes, representing a critical gap in the literature.

In the present study, we aimed to determine the prevalence of RLS in a real-world cohort of CML patients receiving TKI therapy at a tertiary referral center. We also sought to evaluate potential associations between RLS and treatment-related factors, comorbidities, and disease control, as well as the impact of RLS on psychological well-being and functional status, including HRQoL.

## Methods

### Study design and participants

This cross-sectional observational study was conducted at the Istanbul University – Cerrahpaşa, Cerrahpaşa Faculty of Medicine. Consecutive adult patients with CML who attended the hematology outpatient clinic between January and August 2023 were invited to participate in the study. Medical records and hospital information system data were reviewed. Two additional comparator groups were included: (1) patients diagnosed with iron deficiency anemia (IDA) or iron deficiency (ID), and (2) healthy individuals without any known chronic illnesses undergoing routine blood tests, which were previously included in an institutional study [5].

The inclusion criteria for the CML group were as follows: patients with a confirmed diagnosis of CML, based on the presence of the *BCR::ABL1* fusion gene as detected by PCR analysis, who were actively followed at our center and were alive at the time of enrollment. Exclusion criteria included a history of chronic kidney disease, cerebrovascular events, neurodegenerative diseases (e.g., Parkinson’s disease, multiple sclerosis), prior diagnosis of neuropathy of any etiology, chronic alcohol or substance use, and pregnancy.

### Clinical and laboratory assessments

Demographic characteristics, disease history, European Treatment and Outcome Study long-term survival (ELTS) risk scores, TKI therapy (type and duration), comorbidities, concomitant medications, genetic data, anthropometric measurements, smoking and alcohol use, and laboratory findings (complete blood count, iron studies, renal function, fasting glucose, thyroid function, vitamin B12, and folate levels) were recorded using hospital and national healthcare databases.

The diagnosis of RLS was established according to the International Restless Legs Syndrome Study Group (IRLSSG) diagnostic criteria [8], which include: (1) an urge to move the legs usually accompanied by unpleasant sensations, (2) symptom onset or worsening during rest or inactivity, (3) partial or complete relief with movement, and (4) a clear evening or nighttime predominance. We applied the IRLSSG Rating Scale after the diagnosis was established to assess symptom severity [9] (Supplementary Table 1). The pain and neuropathy assessment included the LANSS Pain Scale [10]. Neurological evaluations included sensory modalities (pain, temperature, vibration, light touch, proprioception) and deep tendon reflex testing.

Comprehensive psychometric evaluations were conducted for all CML participants using validated, widely accepted instruments. Anxiety, depression, and hopelessness were assessed using the Beck Anxiety Inventory (BAI), the Beck Depression Inventory (BDI), and the Beck Hopelessness Scale (BHS), respectively, each administered according to their standardized guidelines [11–13]. Fatigue was measured using the Functional Assessment of Chronic Illness Therapy – Fatigue Scale (FACIT-F) [14], and sleep quality was evaluated with the Pittsburgh Sleep Quality Index (PSQI) [15]. HRQoL was assessed by using both the general cancer-specific European Organisation for Research and Treatment of Cancer Quality of Life Questionnaire (EORTC QLQ-C30, version 3.0) and the CML-specific module (EORTC QLQ-CML24), following EORTC recommendations [16, 17]. All instruments were administered in their validated Turkish versions, following standard cultural adaptation procedures and scoring guidelines [18–26].

### Neurological/electrophysiological evaluation

To minimize diagnostic overlap with nocturnal leg cramps or musculoskeletal symptoms frequently observed during TKI therapy, all patients with suspected RLS underwent a detailed neurological examination, a structured symptom assessment focusing on circadian patterns and relief with movement, a LANSS Pain Scale evaluation, and electrophysiological studies to exclude peripheral neuropathy. Patients with RLS typically describe an urge to move their legs, accompanied by unpleasant, non-painful sensations such as restlessness, tingling, crawling, or pulling. These symptoms characteristically worsened during periods of rest or inactivity, showed a clear evening or nighttime predominance, and were partially or completely relieved by movement. In contrast, nocturnal leg cramps and TKI-related musculoskeletal symptoms were described as painful muscle contractions or persistent musculoskeletal pain, lacked an urge to move, were not consistently relieved by movement, and did not demonstrate a circadian pattern. To exclude peripheral neuropathy, electromyographic and neurological evaluations were performed, including EMG conducted according to a standardized polyneuropathy protocol. Motor and sensory nerve conduction studies were conducted in the nondominant upper limb (median and ulnar nerves) and both lower limbs (peroneal, tibial, sural, and superficial peroneal nerves). In the presence of pathological findings, additional contralateral limb assessments were performed.

Sympathetic skin response (SSR) testing was performed in a controlled, quiet setting with the patient in a supine, relaxed state. Silver/silver-chloride surface electrodes were placed on the palmar and plantar regions. Stimulation was delivered to the contralateral median or tibial nerve, with response latency and amplitude recorded using standard filter and gain settings. SSR responses were analyzed for presence, latency, and amplitude.

### Statistical analysis

Descriptive statistics were presented as frequencies and percentages for categorical variables, and as mean ± standard deviation or median with minimum and maximum values for continuous variables, as appropriate. The normality of distribution was assessed using the Shapiro–Wilk test. For between-group comparisons, the independent samples t-test or Mann–Whitney U test was used for two-group comparisons of continuous variables, while one-way ANOVA or the Kruskal–Wallis test was applied for comparisons involving more than two groups. Categorical variables were analyzed using the chi-square test or Fisher’s exact test, as appropriate. Post hoc pairwise comparisons were conducted with Bonferroni adjustment. Correlation analyses were performed using either Pearson or Spearman’s rank correlation, depending on the distributional properties of the data. To identify independent predictors of RLS, binary logistic regression analysis was employed. Variables with a *p value <* 0.20 in the univariate analysis were included in the multivariate model, which was constructed using the Forward Stepwise method. All statistical analyses were performed using IBM SPSS Statistics for Windows, version 29.0 (IBM Corp., Armonk, NY, USA), with a two-tailed significance threshold set at *p* < 0.05 and 95% confidence intervals.

### Ethical considerations

The Ethics Committee of Istanbul University – Cerrahpaşa, Cerrahpaşa Faculty of Medicine, approved the study protocol (Approval No: E-83045809-604.01.01-593057, Date: January 18, 2023). Written informed consent was obtained from all participants prior to their inclusion in the study. All procedures were conducted in accordance with the ethical standards of the institutional research committee and the 1964 Declaration of Helsinki, as well as its subsequent amendments. For retrospective data analysis, patient records were anonymized and handled in compliance with national data protection regulations.

### Results

### Patient characteristics

A total of 164 adult patients with CML were enrolled between January and August 2023. The median age was 53 years (range, 19–85 years), and the median disease duration was 11 years (range, 2–24 years). Eighty-six patients (52.4%) were male. Most patients were receiving imatinib (78.7%), while others were on second-generation TKIs (20.7%) or ponatinib (0.6%). Based on ELTS risk stratification, 71.4% (*n* = 105), 19.1% (*n* = 28), and 9.5% (*n* = 14) of patients were classified as low-, intermediate-, and high-risk, respectively. At least one comorbidity was present in 75.4% of patients, with hypertension (24.7%) and type 2 diabetes mellitus (9.9%) being the most common ones (Table 1).

**Table 1:** Demographics and treatment characteristics of CML patients

Parameter	Result
Sex, n (%)	
Male	86 (52.4)
Female	78 (47.6)
Median age at diagnosis, years (range)	41 (7–76)
Comorbidity, n (%)	
Present	122 (75.4)
Absent	40 (24.6)
ELTS risk scores [*n* = 147], n (%)	
Low	105 (71.4)
Intermediate	28 (19.1)
High	14 (9.5)
Median TKI exposure, months (range)	149 (20–254)
Current TKI, n (%)	
Imatinib	129 (78.7)
2nd -generation TKI	34 (20.7)
Nilotinib	18 (52.9)
Dasatinib	13 (38.2)
Bosutinib	3 (8.9)
Ponatinib	1 (0.6)

**Fig. 1:**
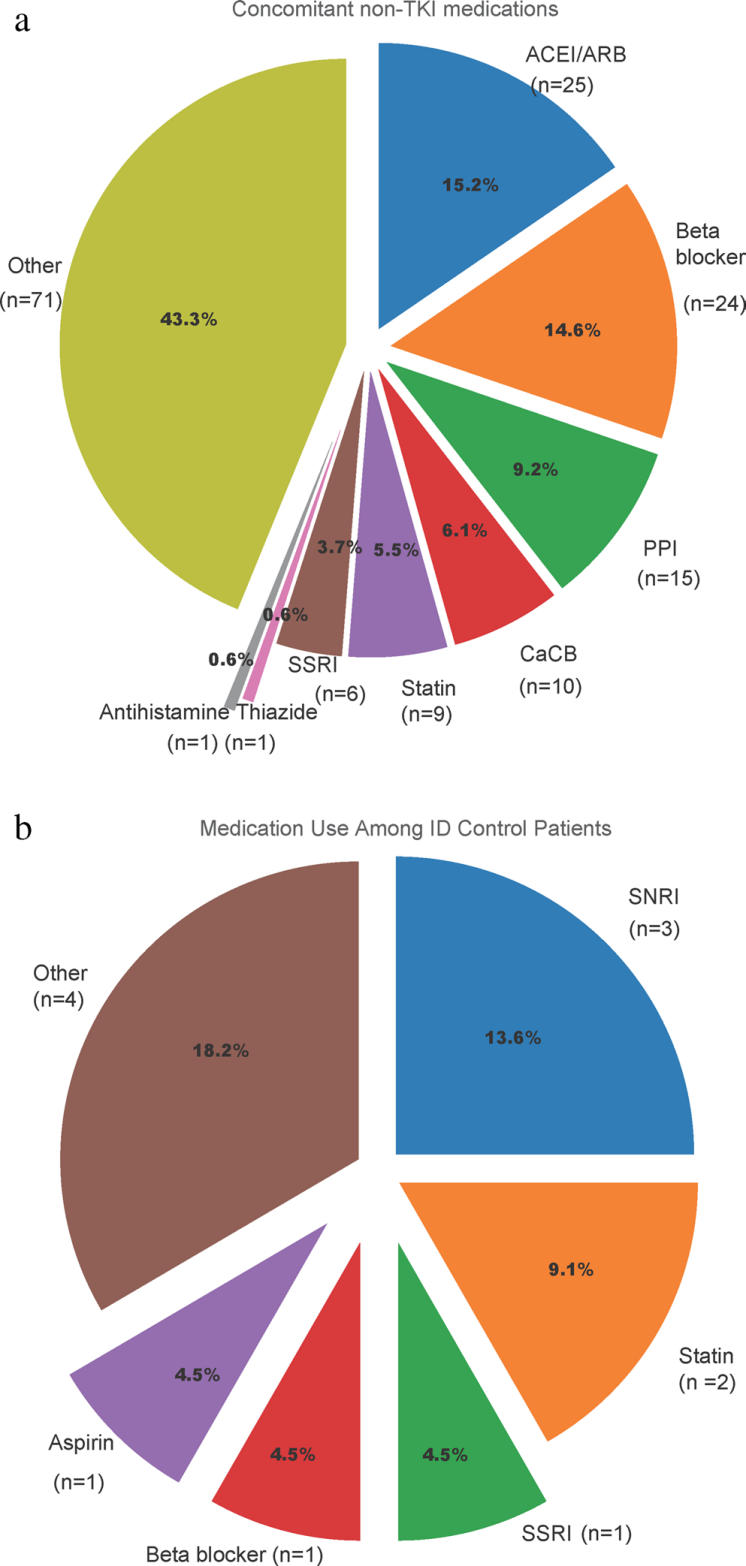
Concomitant non-TKI medications used by patients with chronic myeloid leukemia **a** Distribution of medications among ID control patients **b** **(**ACEI/ARB, angiotensin-converting enzyme inhibitor/angiotensin receptor blocker; CaCB, calcium channel blocker; ID, iron deficiency; PPI, proton pump inhibitor; SNRI, serotonin–norepinephrine reuptake inhibitor; SSRI, selective serotonin reuptake inhibitor; TKI, tyrosine kinase inhibitor)

Concomitant medications in CML patients and ID controls are summarized in Fig. 1a and b. The most frequently used drug classes among CML patients were angiotensin-converting enzyme inhibitors (ACEis) or angiotensin receptor blockers (ARBs) (15.2%), beta-blockers (14.6%), and proton pump inhibitors (9.2%) (Fig. 1a). Among ID controls, serotonergic agents (SNRIs, SSRIs) were the most commonly used concomitant medications, followed by statins and low rates of cardiovascular and miscellaneous drugs (Fig. 1b).

### Prevalence and clinical correlates of restless legs syndrome

RLS was detected in 33 patients (20.1%) with CML. Even after excluding cases with potential secondary causes (e.g., iron deficiency or diabetic neuropathy), the adjusted prevalence of RLS remained substantial at 16.5% (*n* = 27). Most patients with RLS were newly diagnosed (*n* = 30, 90.9%), and symptoms predated the CML diagnosis in 16.7% (*n* = 5) of cases. Among the controls, RLS was observed in 10 out of 22 patients with ID (45.5%), whereas none of the 23 healthy individuals had RLS.

There were no significant differences between RLS-positive and RLS-negative patients in terms of sex, age at diagnosis, smoking, alcohol use, BMI, comorbidities, TKI type and duration, or the distribution of ELTS risk scores (Table 2).

**Table 2:** Comparison of clinical and psychometric parameters between CML patients with and without RLS (^a^t-test, ^b^Chi-Square Test, ^c^Mann-Whitney U Test)

Parameter	RLS (*n* = 33)	Non-RLS (*n* = 131)	*p* value
Female sex, n (%)	19 (57.6)	59 (45.0)	0.200^a^
Median age at diagnosis, years (range)	43 (17–69)	41 (7–73)	0.228^a^
Comorbidity, present (%)	27 (81.8)	94 (71.8)	0.240^b^
ELTS risk scores, n (%)			0.932^b^
Low	22 (73.3)	83 (70.9)	
Intermediate	5 (16.7)	23 (19.7)	
High	3 (10.0)	11 (9.4)	
Median TKI exposure, months (range)	167 (38–250)	141 (20–254)	0.300^c^
Current TKI, n (%)			0.828^b^
Imatinib	25 (75.8)	104 (79.4)	
2nd -generation TKI	8 (24.2)	26 (19.8)	
Nilotinib	6 (75.0)	12 (44.4)	
Dasatinib	1 (12.5)	12 (44.4)	
Bosutinib	1 (12.5)	2 (7.4)	
Ponatinib	0 (0)	1 (0.8)	
PSQI, [poor], n (%)	28 (90.3)	79 (66.4)	**0.016** ^**b**^
Beck Anxiety, [moderate/severe], n (%)	10 (30.3)	7 (5.3)	**< 0.001** ^**b**^
Beck Depression, [moderate/severe], n (%)	9 (27.3)	11 (8.4)	**0.005** ^**b**^
Beck Hopelessness, [moderate/severe], n (%)	9 (27.3)	18 (13.8)	0.117^b^
FACIT-F, [severe fatigue], n (%)	17 (51.5)	20 (15.3)	**< 0.001** ^**b**^
QLQ-C30 global score, mean ± SD	82.49 ± 17.03	83.26 ± 14.66	0.783^c^
CML24 symptom burden, mean ± SD	40.4 ± 19.2	20.7 ± 15.3	**< 0.001** ^**c**^
CML24 mood/worry, mean ± SD	36.36 ± 19.96	18.38 ± 19.47	**< 0.001** ^**c**^
CML24 daily interference, mean ± SD	39.72 ± 23.26	18.39 ± 18.37	**< 0.001** ^**c**^
CML24 care satisfaction, mean ± SD	66.2 ± 24.1	66.9 ± 27.3	0.751^c^
CML24 body image dissatisfaction, mean ± SD	34.34 ± 28.25	16.78 ± 23.52	**< 0.001** ^**c**^
CML24 social satisfaction, mean ± SD	57.6 ± 22.5	60.8 ± 29.4	**0.487** ^**c**^

### TKI use, dose adjustments, and molecular responses

TKI type (imatinib vs. second-generation) was not significantly associated with the presence of RLS, nor were TKI exposure durations. No significant differences were observed in sleep quality, anxiety, depression, or HRQoL scores between patients using first-generation versus second-generation TKIs, except for higher care satisfaction in those receiving second-generation TKIs (*p* = 0.030).

Dose reduction due to adverse events was observed in 18.2% of RLS-positive patients and 17.6% of RLS-negative patients (*p* = 0.933). The molecular response status did not differ significantly between the groups. The percentages of patients with MMR and DMR were similar between RLS-positive and RLS-negative patients (12.1% vs. 18.3%, *p* = 0.196, and 87.9% vs. 70.2%, *p* = 0.196, respectively).

### Comparisons with control groups

CML patients with RLS were significantly older and had higher BMI compared to both RLS-negative CML patients and controls (*p* = 0.004 and *p* = 0.006, respectively). Hematologic indices, including RBC count, hemoglobin, hematocrit, MCV, RDW, and platelet count, showed significant differences across groups (*p* < 0.001), with ID controls generally demonstrating lower hemoglobin and iron parameters (Supplementary Table 2).

Biochemical parameters, including serum creatinine, glucose, TSH, magnesium, and transferrin saturation, also differed significantly between groups (all *p* < 0.05). RLS-positive CML patients had higher fasting glucose and creatinine levels, while ID controls had lower ferritin and transferrin saturation. Apart from the noted findings, there were no significant biochemical differences between CML patients with RLS, ID controls, and healthy individuals (*p* = NS) (Supplementary Table 3).

EMG-confirmed polyneuropathy was present in 22.2% of RLS-positive patients, all of whom had type 2 diabetes. No polyneuropathy was detected in the control groups.

### Psychometric and Quality of Life Evaluations

When compared to CML patients without RLS, those with RLS exhibited significantly poorer patient-reported outcomes. Specifically, RLS-positive patients demonstrated markedly worse sleep quality (90.3% vs. 66.4%, *p* = 0.016), higher levels of anxiety (*p* < 0.001), elevated depression scores (*p* = 0.005), and greater fatigue (*p* < 0.001). However, no significant difference in hopelessness scores was observed between the two groups (*p* = 0.117). Furthermore, on the EORTC QLQ-CML24 and QLQ-C30 assessments, patients with RLS reported a higher overall symptom burden (mean score: 40.4 vs. 19.8, *p* < 0.001) as well as significantly impaired emotional functioning, increased interference with daily life activities, and more negative body image perceptions (all *p* < 0.001). In contrast, no statistically significant differences were identified in satisfaction with care or levels of social functioning (Table 2).

### Multivariate Predictors of RLS

Although age, BMI, hypertension, beta-blocker use, ACEi/ARB use, PSQI score, FACIT-Fatigue score, and multiple CML24 domains (including Worry/Mood Impact, Symptom Burden, Daily Life, and Body Image) were found to be significantly associated with the presence of RLS in univariate analyses, multivariate binary logistic regression analysis identified two independent predictors for RLS as ACEi/ARB use and CML24 Symptom Burden score (Table 3). The final model, generated using the Forward Stepwise method, demonstrated an explanatory power of 38% (Nagelkerke R² = 0.38). According to this model, not using ACEi/ARB was associated with a 0.235-fold reduced risk of RLS compared to users of ACEi/ARB. Additionally, each one-point increase in the CML24 Symptom Burden score was associated with a 1.077-fold higher likelihood of RLS (Table 3).

**Table 3:** Univariate and multivariate logistic regression analysis of risk factors associated with the development of RLS in patients with CML

	Univariate Binary Logistic Regression Analysis				Multivariate Binary Logistic Regression Analysis			
	*p* value	Odds Ratio	95% Confidence Interval		*p* value	Odds Ratio	95% Confidence Interval	
			Lower	Upper			Lower	Upper
Sex	0.200	0.604	0.279	1.306				
Age	**0.046**	1.031	1.000	1.063				
BMI	**0.044**	1.091	1.002	1.187				
Smoking	0.616							
Alcohol Consumption	0.974	0.982	0.339	2.847				
Age at Diagnosis	0.255	1.017	0.988	1.048				
Follow-up Duration	**0.058**	1.067	0.998	1.142				
Duration of TKI Exposure	0.450	1.003	0.996	1.010				
DM	**0.123**	2.377	0.791	7.139				
Hypertension	**0.029**	2.480	1.096	5.612				
Arrhythmia	0.449	0.552	0.119	2.564				
Other Comorbidities	0.459	1.335	0.621	2.874				
History of Surgery	0.567	1.261	0.569	2.794				
Imatinib	0.877							
Prior Use of Imatinib	0.720	0.750	0.155	3.623				
Current Use of Imatinib	0.608	0.728	0.216	2.449				
Type of TKI Used	0.649	1.233	0.500	3.037				
Need for TKI Dose Reduction	0.766	0.859	0.316	2.336				
Calcium Channel Blocker	0.450	0.444	0.054	3.645				
Beta Blocker	**0.023**	2.974	1.162	7.613				
ACE Inhibitor or ARB	**0.006**	3.619	1.434	9.133	**0.010**	0.235	0.077	0.712
Proton Pump Inhibitor	**0.160**	2.288	0.721	7.259				
Other Medications	**0.194**	1.687	0.766	3.713				
Current *BCR::ABL1* Level	0.501	0.015	0.000	3217.534				
White Blood Cell Count	0.449	1.080	0.885	1.318				
Red Blood Cell Count	0.456	0.798	0.441	1.444				
Hemoglobin	0.317	0.890	0.708	1.118				
Hematocrit	0.343	0.963	0.890	1.041				
Mean Corpuscular Volume	0.933	0.997	0.939	1.059				
Red Cell Distribution Width	0.815	1.037	0.766	1.404				
Platelet Count	0.430	1.003	0.996	1.009				
PSQI	**0.015**	4.726	1.354	16.493				
Beck Hopelessness Scale	**0.118**							
Beck Hopelessness Scale/ 1	0.117	2.051	0.834	5.043				
Beck Hopelessness Scale/ 2	0.123	2.564	0.775	8.484				
Beck Hopelessness Scale/ 3	0.047	4.103	1.017	16.542				
Functional Assessment of Chronic Illness Therapy – Fatigue	**< 0**.**001**	5.897	2.565	13.555				
QLQC30 Summary Score	0.793	0.997	0.972	1.022				
QLQ-cml24 Symptom Burden	**< 0**.**001**	1.065	1.038	1.093	**< 0**.**001**	1.077	1.044	1.111
QLQ-cml24 Worry/Mood Impact	**< 0**.**001**	1.039	1.020	1.058				
QLQ-cml24 Daily Life Impact	**< 0**.**001**	1.048	1.027	1.070				
QLQ-cml24 Satisfaction with Care	0.963	1.000	0.987	1.014				
QLQ-cml24 Body Image	**0.001**	1.025	1.010	1.040				
QLQ-CML24 Social Life Satisfaction	0.554	0.996	0.983	1.010				
ELTS Score	0.858	0.923	0.385	2.214				
Low Risk	0.932							
Intermediate Risk	0.718	0.820	0.280	2.404				
High Risk	0.967	1.029	0.264	4.010				

## Discussion

To our knowledge, this is the first study to evaluate the prevalence and clinical implications of RLS in patients with CML. While prior reports have demonstrated increased RLS frequency in *BCR::ABL1*-negative MPNs [5–7], no studies have systematically assessed RLS in CML.

Accordingly, we employed a comprehensive battery of validated psychometric and clinical assessment tools, including standardized questionnaires for mood, fatigue, sleep quality, and HRQoL. Electromyographic and neurological evaluations were also conducted to exclude peripheral neuropathy. By utilizing this multimodal psychometric, neurologic, and clinical approach, our study aimed not only to elucidate the burden, prevalence, and clinical relevance of RLS in this unique patient population but also to raise awareness among clinicians regarding the importance of RLS screening in the long-term management of CML.

In our cohort, the prevalence of RLS among CML patients was 20.1%, substantially higher than the general population rates reported both globally (7.2–11.5%) [27–29] and within Turkey (3.2–5.5%) [30–33]. Notably, this prevalence persisted even after excluding patients with well-known secondary causes of RLS, including chronic kidney disease, DM, ID, and neurological disorders. This suggests a possible intrinsic link between CML pathophysiology and the development of RLS.

Comparable frequencies of RLS have also been reported in other *BCR::ABL1*-negative MPNs. In an independent cohort, RLS was identified in 25.9% of patients with polycythemia vera (PV, *n* = 27) and 34.8% of those with essential thrombocythemia (ET, *n* = 23) [5]. These findings suggest that the elevated prevalence of RLS may be a broader feature of MPNs, potentially implicating shared pathogenic pathways such as systemic inflammation, microvascular dysfunction, or dopaminergic dysregulation. Although TKIs have previously been implicated in rare cases of neuropathy [34], we found no evidence that RLS was associated with either the type of TKI or treatment duration. Furthermore, none of the patients discontinued TKI therapy due to RLS-related symptoms. Interestingly, molecular response rates were similar between RLS-positive and RLS-negative patients, with DMR achieved in over 70% of both groups. This supports the view that RLS does not compromise disease control in CML. These results suggest that the presence of RLS is not associated with inferior molecular outcomes or increased toxicity requiring TKI dose modifications.

Nevertheless, RLS exerted a substantial negative impact on the HRQoL of patients. CML patients with RLS reported significantly higher levels of anxiety, depression, and fatigue, worse sleep quality, and greater symptom burden, emotional distress, and body image concerns. These findings echo meta-analyses in non-CML populations, where RLS has been linked with deteriorated HRQoL [35]. Our multivariate analysis further revealed that an increased symptom burden independently predicted the presence of RLS in CML patients (OR = 1.077). These findings highlight that RLS may define a distinct subgroup of CML patients experiencing disproportionately impaired quality of life, superimposed on the already measurable burden reported in TKI-treated populations.

Electrophysiological evaluations revealed that 22.2% of RLS-positive patients had evidence of peripheral polyneuropathy, all of whom also had comorbid type 2 DM. This observation underscores the importance of thorough neurological assessments in distinguishing RLS from symptoms associated with diabetic peripheral neuropathy. Although RLS and diabetic neuropathy may co-occur, careful distinction is essential given their overlapping symptomatology and differing pathophysiological mechanisms.

We also compared RLS severity and associated features between CML patients and a control group with ID. Although iron parameters were lower in the ID group, RLS severity scores were similar between the two cohorts, suggesting that RLS in CML may be as symptomatic as in iron deficiency-related RLS despite different etiologies. This further strengthens the need to recognize and manage RLS in CML independently from classical causes.

In this cohort, most patients with RLS were newly diagnosed at the time of the study, and no standardized pharmacological treatment for RLS had been initiated prior to assessment. Following diagnosis, patients were informed about RLS and its potential contributing factors, reversible causes were addressed when applicable, and referral to neurology was offered for further evaluation and management.

Hypertension emerged as another interesting factor, although no consistent association with RLS has been demonstrated in the literature [36, 37]. Our single-variable analysis showed an increased risk of RLS in hypertensive patients, and multivariable regression revealed that not using ACEis or ARBs was associated with a reduced risk of RLS. These findings warrant further exploration regarding the role of autonomic dysregulation and vascular factors in CML-associated RLS.

Finally, while no prior literature has specifically linked RLS to CML, parallels may be drawn from studies in other MPNs. Whether similar processes exist in CML remains speculative and necessitates mechanistic investigations.

## Limitations

This study has several limitations that must be acknowledged. First, its cross-sectional and retrospective design precludes causal inference regarding the relationship between CML and the development of RLS. Longitudinal data would be required to establish temporal associations or evaluate the long-term impact of RLS on treatment adherence and disease course. Second, although strict exclusion criteria and objective assessments such as EMG and the LANSS scale were applied to rule out peripheral neuropathy and other mimicking conditions, the diagnosis of RLS was still partially reliant on patient-reported symptoms, which may introduce reporting bias. Information regarding symptom onset prior to CML diagnosis was obtained retrospectively based on patient recall and medical records, and is therefore subject to recall bias. Third, all patients included in the study were receiving TKIs; thus, the lack of a treatment-naïve control group or patients undergoing treatment-free remission (TFR) limits the ability to fully disentangle disease-related versus treatment-related contributors to RLS. Fourth, although polysomnographic evaluation is considered the gold standard for the objective assessment of sleep disorders, PSG data were not collected in this study. This represents a methodological limitation that may have restricted a more detailed characterization of sleep disturbances associated with RLS in CML. However, PSG is generally not required for the clinical diagnosis of RLS, which is primarily based on standardized diagnostic criteria [4]. Finally, while we assessed multiple psychosocial and clinical outcomes, the lack of neuroimaging or mechanistic biomarkers hinders pathophysiological insights into the association between CML and RLS. Given the cross-sectional design and the reliance on patient-reported outcomes, these findings do not allow causal inference, and the observed associations should be interpreted with caution. The impact of RLS on HRQoL may partly overlap with the general symptom burden associated with long-term TKI therapy. Therefore, the present findings should be considered hypothesis-generating rather than definitive and warrant confirmation in prospective longitudinal studies.

## Conclusion

With the chronic nature of CML in most patients with optimal responses under TKI therapy, patients often achieve long-term survival and preserved functional capacity. Our study identified RLS as a previously unrecognized comorbidity in patients with CML, with a prevalence significantly higher than in the general population. This association persisted even after excluding classical secondary causes of RLS, including ID, diabetic neuropathy, and chronic kidney disease. While known risk factors such as ID or diabetic neuropathy were not differentially represented, patients with RLS demonstrated significantly worse sleep quality, higher levels of anxiety and depression, and increased fatigue, ultimately translating into impaired HRQoL. These were independent of age, sex, CML risk stratification, and the type or duration of TKI therapy. These findings suggest that RLS in CML may arise from disease-specific mechanisms independent of TKIs or classical RLS risk factors.

While no prior studies have directly linked RLS to CML, analogous pathophysiological mechanisms have been proposed in other MPNs, including increased blood viscosity, platelet activation, and microvascular dysregulation. Whether similar processes, potentially mediated by leukocytosis or proinflammatory cytokine activity, contribute to RLS in CML remains to be elucidated.

Importantly, RLS did not adversely affect hematological outcomes, including molecular response or tolerability of the TKI dose. Nevertheless, the syndrome represents a substantial burden of symptoms. Given the lack of previous studies investigating this association, our work serves as the first to highlight this clinical entity in CML. Routine screening for RLS symptoms in CML patients, regardless of iron status, may be warranted. Further prospective, mechanistic studies are essential to better characterize the pathogenesis of RLS in this population and to develop targeted supportive interventions aimed at improving patient-centered outcomes.

Furthermore, exploration of RLS dynamics in patients achieving treatment-free remission may offer novel insights into the reversibility and disease dependence of this comorbidity. Recognizing and addressing RLS may represent a meaningful step toward improving HRQoL in CML beyond molecular remission.

## Supplementary Information

Below is the link to the electronic supplementary material.

Supplementary Material 1

Supplementary Material 2

Supplementary Material 3

## Data Availability

Data supporting the findings of this study are available from the corresponding author upon reasonable request.

## References

[1] Arber DA, Orazi A, Hasserjian R, Errata (2016) The 2016 revision to the World Health Organization classification of myeloid neoplasms and acute leukemia (Blood (2016) 127, 20 (2391-2405)). Blood Am Soc Hematol 128:462–3 10.1182/BLOOD-2016-06-721662

[2] Nangalia J, Green AR (2017) Myeloproliferative neoplasms: From origins to outcomes. Blood Am Soc Hematol 130:2475–2483. 10.1182/BLOOD-2017-06-782037 10.1182/blood-2017-06-782037 29212804

[3] Goldman JM (2010) Chronic Myeloid Leukemia: A Historical Perspective. Semin Hematol 47:302–311. 10.1053/j.seminhematol.2010.07.001 20875546 10.1053/j.seminhematol.2010.07.001

[4] Manconi M, Garcia-Borreguero D, Schormair B, Videnovic A, Berger K, Ferri R et al (2021) Restless legs syndrome. Nat Rev Dis Primers Nat Res. 7 10.1038/S41572-021-00311-Z 10.1038/s41572-021-00311-z 34732752

[5] Seyhan Erdoğan D, Benbir Şenel G, Gündüz A, Uçar BP, Elverdi T, Salihoğlu A et al (2023) A cross-sectional study on restless legs syndrome (RLS) in polycythemia Vera (PV): is iron deficiency the only culprit? Neurol res, vol 45. Taylor and Francis Ltd., pp 1144–1151. 10.1080/01616412.2023.2257443 10.1080/01616412.2023.2257443 37736879

[6] Tobiasson M, Alyass B, Söderlund S, Birgegård G (2010) High prevalence of restless legs syndrome among patients with polycytemia Vera treated with venesectio. Med Oncol 27:105–107. 10.1007/S12032-009-9180-5 19225914 10.1007/s12032-009-9180-5

[7] Scherber RM, Kosiorek HE, Senyak Z, Dueck AC, Clark MM, Boxer MA et al (2016) Comprehensively Understanding fatigue in patients with myeloproliferative neoplasms. Cancer vol 122. John Wiley and Sons Inc., pp 477–485. 10.1002/CNCR.29753 10.1002/cncr.29753 26670597

[8] Allen RP, Picchietti DL, Garcia-Borreguero D, Ondo WG, Walters AS, Winkelman JW et al (2014) Restless legs syndrome/Willis-Ekbom disease diagnostic criteria: updated international restless legs syndrome study group (IRLSSG) consensus criteria - history, rationale, description, and significance. Sleep Med Elsevier B V 15:860–873. 10.1016/j.sleep.2014.03.025 10.1016/j.sleep.2014.03.025 25023924

[9] Validation of the International Restless Legs Syndrome Study Group rating scale for restless legs syndrome (2003) Sleep Med 4:121–132. 10.1016/S1389-9457(02)00258-7 10.1016/s1389-9457(02)00258-7 14592342

[10] Bennett M, The, LANSS Pain Scale (2001) : The Leeds assessment of neuropathic symptoms and signs. Pain 92:147–157. 10.1016/S0304-3959(00)00482-6 11323136 10.1016/s0304-3959(00)00482-6

[11] Beck AT, Weissman A, Lester D, Trexler L (1974) The measurement of pessimism: the hopelessness Scale. J Consult Clin Psychol 42:861–865. 10.1037/H0037562 4436473 10.1037/h0037562

[12] Beck AT, Steer RA, Ball R, Ranieri WF (1996) Comparison of Beck depression inventories -IA and -II in psychiatric outpatients. J Pers Assess 67:588–597. 10.1207/S15327752JPA6703_13 8991972 10.1207/s15327752jpa6703_13

[13] Beck AT, Epstein N, Brown G, Steer RA (1988) An inventory for measuring clinical anxiety: Psychometric properties, vol 56. American Psychological Association (APA). J Consult Clin Psychol pp 893–897. 10.1037//0022-006X.56.6.893 10.1037//0022-006x.56.6.893 3204199

[14] Webster K, Cella D, Yost K (2003) The functional assessment of chronic illness therapy (FACIT) measurement system: properties, applications, and interpretation. Health Qual Life Outcomes. 1:79. 10.1186/1477-7525-1-79 10.1186/1477-7525-1-79 PMC317391 14678568

[15] Buysse DJ, Reynolds CF, Monk TH, Berman SR, Kupfer DJ (1989) The Pittsburgh sleep quality index: A new instrument for psychiatric practice and research [Índice de calidad del sueño de Pittsburgh: un nuevo instrumento para la práctica y la investigación psiquiátrica]. Psychiatry Res. Elsevier 28:193–213. https://pubmed.ncbi.nlm.nih.gov/2748771/. Accessed 14 Jul 2025 10.1016/0165-1781(89)90047-4 2748771

[16] Nolte S, Liegl G, Petersen MA, Aaronson NK, Costantini A, Fayers PM et al (2019) General population normative data for the EORTC QLQ-C30 health-related quality of life questionnaire based on 15,386 persons across 13 European countries, Canada and the Unites States. Eur J Cancer. Elsevier Ltd 107:153–63. 10.1016/j.ejca.2018.11.024 10.1016/j.ejca.2018.11.024 30576971

[17] Efficace F, Iurlo A, Patriarca A, Stagno F, Bee PC, Ector G et al (2021) Validation and reference values of the EORTC QLQ-CML24 questionnaire to assess health-related quality of life in patients with chronic myeloid leukemia. Leuk lymphoma, vol 62. Taylor and Francis Ltd., pp 669–678. 10.1080/10428194.2020.1838509 10.1080/10428194.2020.1838509 33153355

[18] Yucel A, Senocak M, Orhan EK, Cimen A, Ertas M (2004) Results of the Leeds assessment of neuropathic symptoms and signs pain scale in turkey: A validation study. Journal of pain, vol 5. Churchill Livingstone Inc., pp 427–432. 10.1016/j.jpain.2004.07.001 10.1016/j.jpain.2004.07.001 15501424

[19] Ay E, Yılmaz NH, Düz ÖA, Özer FF (2019) The Turkish Version of The International Restless Legs Syndrome Study Group Rating Scale Validity and Reliability of. Acta Medica Alanya. Alanya Alaaddin Keykubat Üniversitesi; 3:105–10. 10.30565/MEDALANYA.453150

[20] Agargün MY, Kara H, Anlar O et al (1996) Pittsburgh Uyku Kalitesi Indeksinin Geçerligi Ve Güvenirligi. Türk Psikiyatri Dergisi, 7, 107–115. - References - Scientific Research Publishing. https://www.scirp.org/reference/referencespapers?referenceid=3216586. Accessed 13 Jul 2025

[21] Bostan H, Toptas T, Tanrikulu FP, Kut K, Arikan F, Yilmaz F et al (2020) Quality of Life and Symptom Burden With First- and Second-generation Tyrosine Kinase Inhibitors in Patients With Chronic-phase Chronic Myeloid Leukemia, vol 20. Elsevier Inc., Clin Lymphoma Myeloma Leuk pp 836–842. 10.1016/j.clml.2020.08.009 10.1016/j.clml.2020.08.009 32958432

[22] Guzelant A, Goksel T, Ozkok S, Tasbakan S, Aysan T, Bottomley A (2004) The European organization for research and treatment of cancer QLQ-C30: an examination into the cultural validity and reliability of the Turkish version of the EORTC QLQ-C30. Eur J Cancer Care (Engl) 13:135–144. 10.1111/J.1365-2354.2003.00435.X 15115469 10.1111/j.1365-2354.2003.00435.x

[23] Kronik Hastalık Tedavisi Fonksiyonel Değerlendirmesi Yorgunluk (FACIT Yorgunluk) Ölçeği | TOAD. https://toad.halileksi.net/olcek/kronik-hastalik-tedavisi-fonksiyonel-degerlendirmesi-yorgunluk-facit-yorgunluk-olcegi/. Accessed 13 Jul 2025

[24] Beck Depresyon Envanteri | TOAD. https://toad.halileksi.net/olcek/beck-depresyon-envanteri/. Accessed 13 Jul 2025

[25] [PDF], BECK UMUTSUZLUK ÖLÇEĞİ GEÇERLİLİK ÇALIŞMASI* https://www.acarindex.com/pdfs/289422. Accessed 13 Jul 2025

[26] (PDF) Turkish Version of the Beck Anxiety Inventory Psychometric Properties https://www.researchgate.net/publication/233792003_Turkish_Version_of_the_Beck_Anxiety_Inventory_Psychometric_Properties. Accessed 13 Jul 2025

[27] Yeh P, Walters AS, Tsuang JW (2012) Restless legs syndrome: A comprehensive overview on its epidemiology, risk factors, and treatment. Sleep Breath 16:987–1007. 10.1007/S11325-011-0606-X 22038683 10.1007/s11325-011-0606-x

[28] Fereshtehnejad SM, Shafieesabet M, Shahidi GA, Delbari A, Lökk J (2015) Restless legs syndrome in patients with parkinson’s disease: A comparative study on prevalence, clinical characteristics, quality of life and nutritional status. Acta Neurol Scand Acta Neurol Scand 131:211–218. 10.1111/ANE.12307 25263328 10.1111/ane.12307

[29] Silber MH, Buchfuhrer MJ, Earley CJ, Koo BB, Manconi M, Winkelman JW et al (2021) The Management of Restless Legs Syndrome: An Updated Algorithm, vol 96. Elsevier Ltd, Mayo Clin Proc pp 1921–1937. 10.1016/J.MAYOCP.2020.12.026 10.1016/j.mayocp.2020.12.026 34218864

[30] Sevim S, Dogu O, Çamdeviren H, Bugdayci R, Sasmaz T, Kaleagasi H et al (2003) Unexpectedly low prevalence and unusual characteristics of RLS in Mersin, Turkey. Neurology, vol 61. Lippincott Williams and Wilkins, pp 1562–1569. 10.1212/01.WNL.0000096173.91554.B7 10.1212/01.wnl.0000096173.91554.b7 14663043

[31] Taşdemir M, Erdoǧan H, Börü ÜT, Dilaver E, Kumaş A (2010) Epidemiology of restless legs syndrome in Turkish adults on the Western black sea Coast of turkey: A door-to-door study in a rural area. Sleep Med 11:82–86. 10.1016/J.SLEEP.2008.10.008 19403331 10.1016/j.sleep.2008.10.008

[32] Cakmak VA, Koc B, Nuhoglu I, Topbas M, Ucuncu SY, Deger O et al (2015) Prevalence of restless legs syndrome in trabzon in the Northeast black sea region of turkey: Co-morbidities, socioeconomic factors and biochemical parameters. Neurol res, vol 37. Taylor and Francis Ltd., pp 763–773. 10.1179/1743132815Y.0000000058 10.1179/1743132815Y.0000000058 26001093

[33] Yilmaz NH, Akbostanci MC, Oto A, Aykac O (2013) Prevalence of restless legs syndrome in Ankara, Turkey: An analysis of diagnostic criteria and awareness. Acta Neurol Belg. Springer-Verlag Italia s.r.l.; 113:247–51. 10.1007/S13760-012-0153-7 10.1007/s13760-012-0153-7 23111781

[34] Kavanagh S, Bril V, Lipton JH (2018) Peripheral neuropathy associated with Imatinib therapy for chronic myeloid leukemia. Blood Res KSH 53:172–174. 10.5045/BR.2018.53.2.172 10.5045/br.2018.53.2.172 PMC6021569 29963528

[35] Broström A, Alimoradi Z, Odzakovic E, Kaldo V, Jernelöv S, Lind J et al (2024) Quality of life among patients with restless legs syndrome: A systematic review and meta-analysis. J Clin Neurosci Churchill Livingstone 122:80–91. 10.1016/j.jocn.2024.02.027 10.1016/j.jocn.2024.02.027 38489955

[36] Cholley-Roulleau M, Chenini S, Béziat S, Guiraud L, Jaussent I, Dauvilliers Y (2017) Restless legs syndrome and cardiovascular diseases: A case-control study. PLoS One Public Libr Sci 12 10.1371/JOURNAL.PONE.0176552 10.1371/journal.pone.0176552 PMC5406016 28445539

[37] Walters AS, Rye DB (2009) Review of the relationship of restless legs syndrome and periodic limb movements in sleep to hypertension, heart disease, and stroke. Sleep Am Acad Sleep Med 32:589–597. 10.1093/SLEEP/32.5.589 10.1093/sleep/32.5.589 PMC2675893 19480225

