# Supplementary material for: Restless legs syndrome in chronic myeloid leukemia: an overlooked condition with a significant impact on health-related quality of life
Source: Ann Hematol. 2026 Mar 19;105(4):190. doi: 10.1007/s00277-026-06832-5 (PMC12999824; doi:10.1007/s00277-026-06832-5)
Supplement: Supplementary file 1 — Supplementary Material 1 [file 277_2026_6832_MOESM1_ESM.docx]

**Supplementary Table 1.** The validated Turkish version of the International Restless Legs Syndrome Study Group (IRLSSG) diagnostic criteria and severity rating scale [8,9,19].

**
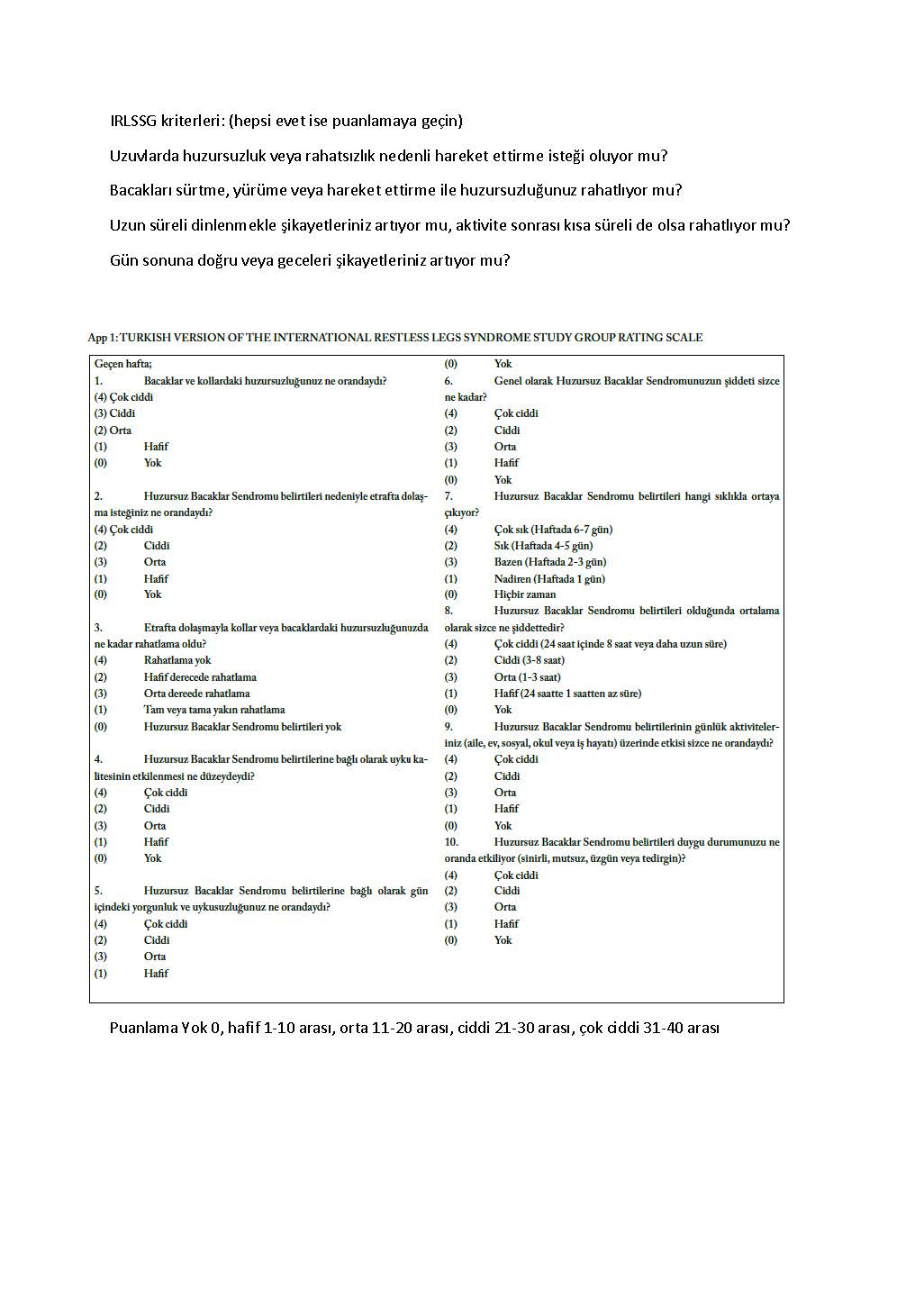
**
