# Supplementary material for: Restless legs syndrome in chronic myeloid leukemia: an overlooked condition with a significant impact on health-related quality of life
Source: Ann Hematol. 2026 Mar 19;105(4):190. doi: 10.1007/s00277-026-06832-5 (PMC12999824; doi:10.1007/s00277-026-06832-5)
Supplement: Supplementary file 2 — Supplementary Material 2 [file 277_2026_6832_MOESM2_ESM.docx]

**Supplementary Table 2.** Comparison of age, body mass index, and complete blood count parameters among CML patients with RLS, CML patients without RLS, iron deficiency (ID) control group, and healthy controls (^a^Kruskal-Wallis Test).

| Parameter | CML patients with RLS  (n=33) | CML patients without RLS  (n=130) | ID Group  (n=22) | Healthy Control Group  (n=23) | *p* value^a^ |
| --- | --- | --- | --- | --- | --- |
| Age, years  Mean ± SD  Median (range) | 58 ± 13,29  60 (31-80) | 52,82 ± 13,24  52 (19-85) | 46 ± 10,96  45 (26-76) | 49,91 ± 8,43  52 (34-63) | **0,004** |
| BMI, kg/m^2^  Mean ± SD  Median (range) | 29,14 ± 4,83  28,65 (20,27-41,33) | 27,35 ± 4,36  27,76 (16,80-41,52) | 25,53 ± 3,19  24,50 (19,40-32,50) | 25,98 ± 3,44  25,60 (20,30-37,40) | **0,006** |
|  | **(n=32)** | **(n=125)** | **(n=22)** | **(n=23)** |  |
| WBC, 10^3/μL  Mean ± SD  Median (range) | 7,12 ± 1,52  7,30 (3-9,30) | 6,83 ± 2,03  6,50 (3,08-12,90) | 6,95 ± 2,05  7,15 (3,30-12,80) | 7,11 ± 1,79  6,90 (4,50-11,10) | **0,488** |
| RBC, 10^6/μL  Mean ± SD  Median (range) | 4,07 ± 0,64  4,07 (2,94-5,26) | 4,17 ± 0,67  4,15 (2,13-6,52) | 4,42 ± 0,45  4,44 (3,17-5,10) | 4,77 ± 0,40  4,80 (3,93-5,30) | **<0,001** |
| HGB, g/dl  Mean ± SD  Median (range) | 12,32 ± 1,60  12,40 (9,50-14,70) | 12,66 ± 1,70  12,80 (6,50-15,90) | 10,91 ± 1,94  10,75 (5,90-14,50) | 41,04 ± 1,11  14,30 (12,20-16,20) | **<0,001** |
| HCT, %  Mean ± SD  Median (range) | 37,18 ± 4,79  38 (28,70-44,30) | 38,10 ± 4,91  38,60 (20-47,90) | 34,15 ± 5,18  34,95 (20-42,20) | 41,57 ± 3,05  41,80 (36,40-47,90) | **<0,001** |
| MCV, fl  Mean ± SD  Median (range) | 91,88 ± 6,14  91,45 (77,40-104,50) | 91,99 ± 6,59  93,00 (66,40-106,20) | 76,98 ± 10,64  80,25 (52,20-97,40) | 86,99 ± 3,56  86 (81,90-93,60) | **<0,001** |
| RDW, %  Mean ± SD  Median (range) | 14,36 ± 1,33  14,10 (12,00-19,70) | 14,30 ± 1,24  14,00 (12,10-19,10) | 16,82 ± 1,89  16,80 (13,70-21,90) | 13,47 ± 0,74  13,40 (12,40-15,20) | **<0,001** |
| PLT, 10^3/μL  Mean ± SD  Median (range) | 252,28 ± 61,43  240,50 (157-463) | 243,39 ± 55,74  235 (115-399) | 305,09 ± 80,02  285,50 (190-523) | 260,96 ± 80,15  232 (173-501) | **0,006** |
