# Supplementary material for: Restless legs syndrome in chronic myeloid leukemia: an overlooked condition with a significant impact on health-related quality of life
Source: Ann Hematol. 2026 Mar 19;105(4):190. doi: 10.1007/s00277-026-06832-5 (PMC12999824; doi:10.1007/s00277-026-06832-5)
Supplement: Supplementary file 3 — Supplementary Material 3 [file 277_2026_6832_MOESM3_ESM.docx]

**Supplementary Table 3.** Comparison of biochemical parameters between CML patients with RLS and control groups (^a^Kruskal-Wallis Test).

| Parameter | CML patients with RLS (n=30) | ID Group  (n=22) | Healthy Control Group  (n=23) | *p* value^a^ |
| --- | --- | --- | --- | --- |
| Urea, mg/dL  Mean ± SD  Median (range) | 32,83 ± 12,20  28,50 (21-73) | 25,95 ± 7,64  26 (17-49) | 26,87 ± 7,03  26 (17-47) | **0,037** |
| Creatinine, mg/dL  Mean ± SD  Median (range) | 0,95 ± 0,25  0,90 (0,61-1,90) | 0,67 ± 0,12  0,63 (0,50-0,91) | 0,77 ± 0,16  0,80 (0,50-1,10) | **<0,001** |
| Fasting Plasma Glucose, mg/dL  Mean ± SD  Median (range) | 107,10 ± 32,29  93,50 (78-226) | 84,73 ± 12,23  83,50 (64-109) | 85,75 ± 10,62  84 (65-104) | **0,001** |
| HBA1C, %  Mean ± SD  Median (range) | 6,04 ± 0,98  5,80 (4,90-8,60) | 5,69 ± 0,46  5,70 (4,70-6,40 | 5,72 ± 0,26  5,80 (5,20-6,10) | 0,855 |
| Magnesium, mEq/L  Mean ± SD  Median (range) | 2,01 ± 0,16  2,01 (1,73-2,31) | 2,14 ± 0,20  2,10 (1,87-2,75) | 2,15 ± 0,19  2,10 (1,83-2,46) | **0,023** |
| TSH, μIU/ml  Mean ± SD  Median (range) | 1,50 ± 1,10  1,21 (0,10-5,57) | 2,38 ± 1,23  2 (0,40-4,80) | 1,84 ± 0,96  1,90 (0,50-4,20) | **0,012** |
| Free T4, ng/dl  Mean ± SD  Median (range) | 1,21 ± 0,19  1,19 (0,91-1,57) | 1,31 ± 0,47  1,17 (0,99-3,18) | 1,35 ± 0,59  1,20 (1-3,60) | 0,983 |
| Free T3, pg/ml  Mean ± SD  Median (range) | 2,89 ± 0,42  2,86 (2,22-3,77) | 3,17±0,67  3,20 (1,20-4,26) | 3,04±0,49  3,13 (1,39-3,68) | **0,038** |
| Vitamin B12, pg/ml  Mean ± SD  Median (range) | 380,47 ± 176,93  374 (120-912) | 370,59 ± 181,63  344 (190-1081) | 395,78 ± 112,69  389 (202-680) | 0,391 |
| Folic acid, μg/dl  Mean ± SD  Median (range) | 8,77 ± 2,59  8,07 (5,28-17,60) | 7,82 ± 4,51  6,10 (2,70-20) | 9,19 ± 3,59  8,27 (5,40-20) | **0,044** |
| Transferrin Saturation, %  Mean ± SD  Median (range) | 21,23 ± 6,39  21 (5-33) | 7,69 ± 5,19  6,50 (0,20-21) | 27,63 ± 7,68  25 (17-46) | **<0,001** |
| Ferritin, μg/dl  Mean ± SD  Median (range) | (n=27)  108,54 ± 86,44  106 (7-327) | (n=22)  15,60 ± 30,51  9,20 (2,50-151) | (n=23)  94,03 ± 62,43  76 (28-269) | **<0,001** |
